# Molecular nonchemically amplified resists based on spirobixanthene backbone: Sulfoxime oxime esters versus sulfonium salts

**DOI:** 10.1002/smo2.70028

**Published:** 2025-11-24

**Authors:** Yu Yan, Chenfei Zhao, Jingwen Hui, Xinfu Zhang, Xue Zhang, Pengzhong Chen, Xiaojun Peng, Yi Xiao

**Affiliations:** ^1^ State Key Laboratory of Fine Chemicals Frontiers Science Center for Smart Materials Oriented Chemical Engineering Dalian University of Technology Dalian China

**Keywords:** e‐beam lithography, molecular glass resist, nonchemically amplified resist, photoresist, spirobixanthene

## Abstract

The nonchemically amplified (nonCA) polymer resists, including ionic and non‐ionic types, have achieved higher resolution and smaller line edge roughness (LER) than traditional chemically amplified ones. However, for polymer resists, chain entanglement is an inevitable limitation for the further reduction of LER. To overcome this problem, it is logical to apply the nonCA concept to molecule‐based resists due to their advantages of monodispersity and small size. To date, only a few examples of ionic sulfonium salts‐based nonCA molecular glass resists (nonCAMGRs) have been reported. They demonstrated high resolution and small LER well, but their electron beam sensitivity seemed less than ideal. To our knowledge, non‐ionic sulfoxime oxime esters‐based molecular resists were not reported yet, which leaves room for new round of more in‐depth reserch on nonCAMGRs. Here, employing the excellent spirobixanthene backbone, we have first designed non‐ionic sulfoxime oxime esters‐based nonCAMGRs X4‐NI‐tf and X4‐NI‐tfb, for comparison, sulfonium salts‐based nonCAMGRs X4‐I‐otfdm was designed. All exhibit favorable thermal properties (T_d,5%_ > 200°C) and film‐forming capabilities (RMSs <0.4 nm). Via EBL, X4‐I‐otfdm achieved higher resolution (16 nm, LER 1.4 nm) than X4‐NI‐tf and X4‐NI‐tfb (20 nm, LER 1.6 nm). But contrast curve revealed that the sensitivity of X4‐NI‐tf and X4‐NI‐tfb (D_100_: 370 and 350 μC/cm^2^) was significantly higher than X4‐I‐otfdm (D_100_: 3300 μC/cm^2^), demonstrating that the sensitivity of sulfoxime oxime esters exceeds that of sulfonium salts and introduction of bromine can further enhance the sensitivity; based on above, X4‐NI‐tfb exhibited the lowest Z‐factor and demonstrated the best overall performance. We believe that nonCAMGRs based on sulfoxime oxime esters represent a strong candidate for high‐performance photoresists.

## Graphical abstract

The sensitivity of sulfoxime oxime esters significantly surpasses that of sulfonium salts in spirobixanthene‐based nonchemically amplified molecular glass resists, and Z‐factor reveals that X4‐NI‐tfb has the best overall lithographic performance.

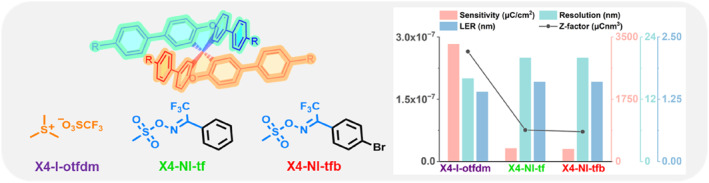

## 1 INTRODUCTION

High‐resolution photoresists are critical to semiconductor manufacturing as the industry approaches the physical limits of Moore's Law,^[1]^ particularly with the adoption of extreme ultraviolet (EUV) lithography. EUV technology enables patterning of sub‐10 nm features, but it demands photoresists capable of overcoming unique challenges such as photon scarcity, resist blur, and stochastic effects.[^2^, ^3^]

Chemically amplified resists (CARs) dominate EUV lithography due to their chemically amplified mechanisms that multiply effectively the impact of each absorbed photon.[^3^, ^4^, ^5^] The amplification enhances sensitivity but introduces critical drawbacks. Inhomogeneous photoacid generator (PAG) distribution and acid diffusion during post‐exposure bake (PEB) exacerbating line edge roughness (LER) and pattern blur undermine resolution.[^6^, ^7^] Some strategies such as PAG bound polymers[^8^, ^9^] are adopted to control acid diffusion, but it is still regrettable to achieve the desired effect with high resolution and low LER.

**FIGURE 1:**
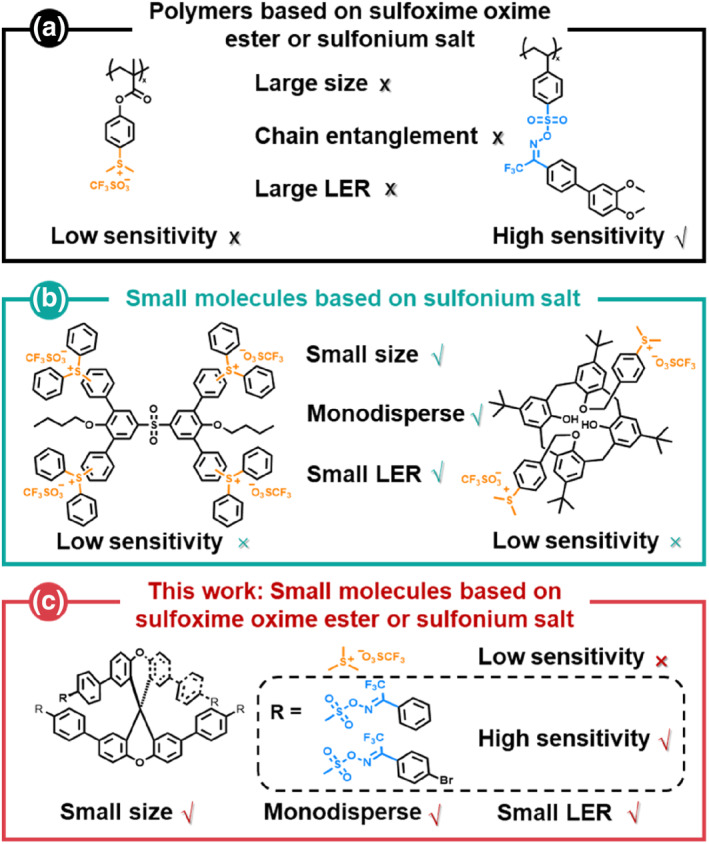
Comparison of high resolution nonCA polymers and small molecules fabricated based on sulfoxime oxime esters and sulfonium salts.[^10^, ^14^, ^18^, ^19^] (a) Characteristics of reported polymers based on sulfoxime oxime ester or sulfonium salt. (b) Characteristics of reported molecular resists based on sulfonium salt. (c) Characteristics of spirobixanthene‐based molecular resists based on sulfoxime oxime ester or sulfonium salt.

Nonchemically amplified resists (nonCARs) rely directly on exposure dose and developer without acid diffusion in CARs; the elimination of photoacid control facilitates the enhancement of resolution. Ionic sulfonium salts and non‐ionic sulfoxime oxime esters have been demonstrated to be EUV‐sensitive by the research groups of Gonsalves and Yang (Figure 1a),[^10^, ^11^, ^12^, ^13^, ^14^] respectively; the resolution of nonchemically amplified polymeric photoresists based on both surpasses that of traditional polymeric CARs; however, polymeric chain entanglement limits the reduction of LER (LER >2.0 nm).

Molecular glass resists (MGRs) are organic small molecules which embrace the characteristics of monodispersity and small volume; these characteristics are inherently conducive to achieving high resolution and low LER due to no interpolymeric chain entanglement. However, the resolution and LER of chemically amplified MGRs still are troubled due to inhomogeneous PAGs distribution and acid diffusion.[^15^, ^16^, ^17^]

Nonchemically amplified MGRs (nonCAMGRs) avoid the use of PAGs, making them a promising option for achieving higher resolution and smaller LER. Yang group reported several ionic nonCAMGRs based on sulfonium salts (Figure 1b),[^18^, ^19^] that achieved ∼14 nm 1:1 line/space (L/S) half pitch (HP) high resolution dense lines and low LER (LER <2.0 nm), but their sensitivity is low (D_100_ > 1000 μC/cm^2^). We have reported excellent spirobixanthene backbone, which exhibits excellent thermal stability and film‐forming capability.^[20]^ Small‐molecule nonCAMGRs based on sulfoxime oxime esters have not yet been reported; utilizing this backbone, we have synthesized non‐ionic nonCAMGRs X4‐NI‐tf and X4‐NI‐tfb for the first time to achieve higher sensitivity (D_100_ = 370 and 350 μC/cm^2^, respectively) (Figure 1c). For comparison, ionic nonCAMGR X4‐I‐otfdm based on sulfonium salts was also synthesized and it exhibited very low sensitivity (D_100_ = 3300 μC/cm^2^). Experimental results demonstrate that the sensitivity of sulfoxime oxime esters surpasses that of sulfonium salts.

## 2 RESULTS AND DISCUSSION

### 2.1 Physical properties of resists

**FIGURE 2:**
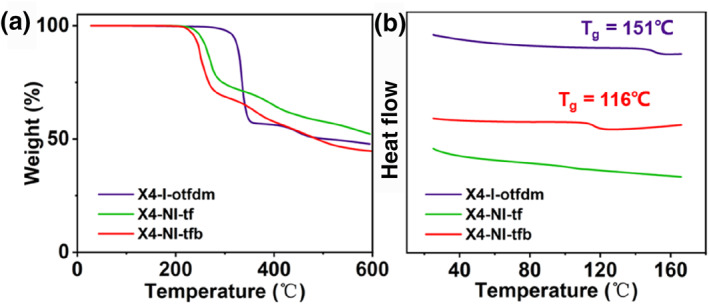
(a) TGA and (b) differential scanning calorimetry (DSC) curves of X4‐I‐otfdm, X4‐NI‐tf and X4‐NI‐tfb.

Thermal decomposition temperature (T_d_) is a critical character for resists; post application bake (PAB) temperature must be lower than the T_d_ to prevent structure deterioration, that ensures the success of subsequent exposure and developing processes. As shown in Figure 2a, thermogravimetry analysis (TGA) reveals T_d,5%_ of X4‐I‐otfdm is 318°C higher than X4‐NI‐tf 251°C and X4‐NI‐tfb 238°C; this result demonstrates that the thermal stability of sulfonium salts surpasses that of sulfoxime oxime esters. Another important temperature is the glass transition temperature (T_g_); the PEB temperature of CARs must be lower than T_g_, but nonCARs based on sulfonium salts and sulfoxime oxime esters give usually up PEB process,[^14^, ^18^] differential scanning calorimetry (DSC) reveals T_g_ of X4‐I‐otfdm and X4‐NI‐tfb is 151°C and 116°C, X4‐NI‐tf has no T_g_ before thermal decomposition (Figure 2b). These favorable thermophysical properties are attributable to the pronounced structural rigidity of spiroxanthene backbone; the thermal analysis confirms that the three resists are stable in the lithography baking process.

### 2.2 Film‐forming properties of resists

**FIGURE 3:**
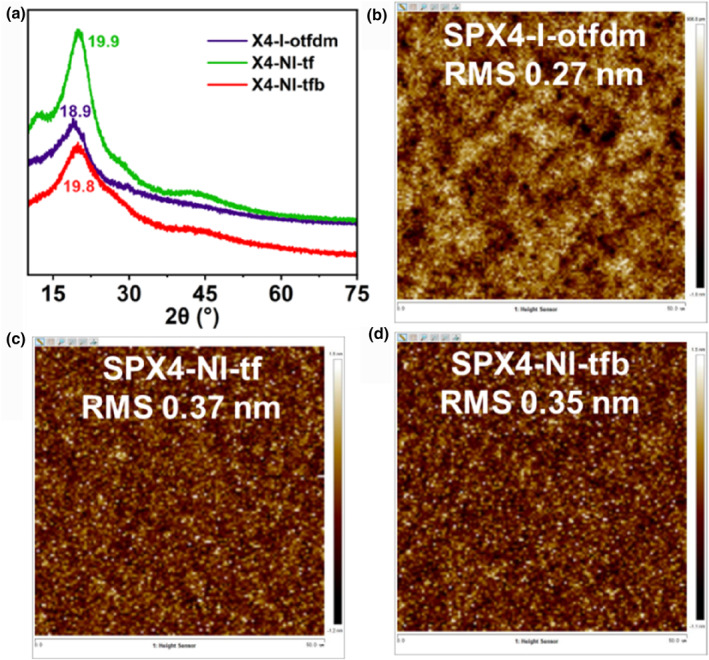
(a) X‐ray diffraction curves and (b–d) AFM images of film surface roughness in 50 μm × 50 μm square area of X4‐I‐otfdm, X4‐NI‐tf and X4‐NI‐tfb.

The amorphous state is the guarantee of smooth film and good pattern morphology,^[21]^ X‐ray diffraction (XRD) displays the board and smooth peaks of X4‐I‐otfdm, X4‐NI‐tf and X4‐NI‐tfb from 10° to 75°, the 2θ value of maximum peak value is observed, respectively, at 18.9°, 19.9° and 19.8° (Figure 3a), that indicates three powders exhibit prefect amorphous stacking, the rigidly distorted spirocyclic architecture of spiroxanthene backbone, when oxygen‐bridged, acts in concert to completely suppress crystallinity, ensuring this characteristic amorphous packing arrangement. A 50 μm × 50 μm square area is scanned by AFM on the film surface; all MGs have good smooth films, the root mean square (RMS) roughness of X4‐I‐otfdm is minimal for 0.27 nm (Figure 3b), and the RMS of X4‐NI‐tf and X4‐NI‐tfb is, respectively, 0.37 and 0.35 nm (Figure 3c,d), it ensures possibility to obtain regular lithography patterns after exposure and development.

### 2.3 Developer and contrast of resists

After exposure, choosing appropriate solvents as developer is essential to form high‐resolution and low LER patterns. As shown in Table S1, X4‐I‐otfdm molecules are ionic salt, which decide it possesses high solubility in polar solvents such as ethyl lactate (EL), 1‐methoxy‐2‐propanol (PGME), isopropyl alcohol (IPA) and H_2_O, X4‐I‐otfdm shows poor solubility in 1‐methoxy‐2‐propyl acetate (PGMEA), 4‐methyl‐2‐pentanone (MIBK) and butyl acetate (BAC). Based on the literature,^[18]^ the mixed solvent of IPA and H_2_O was selected as the developer of X4‐I‐otfdm.

**FIGURE 4:**
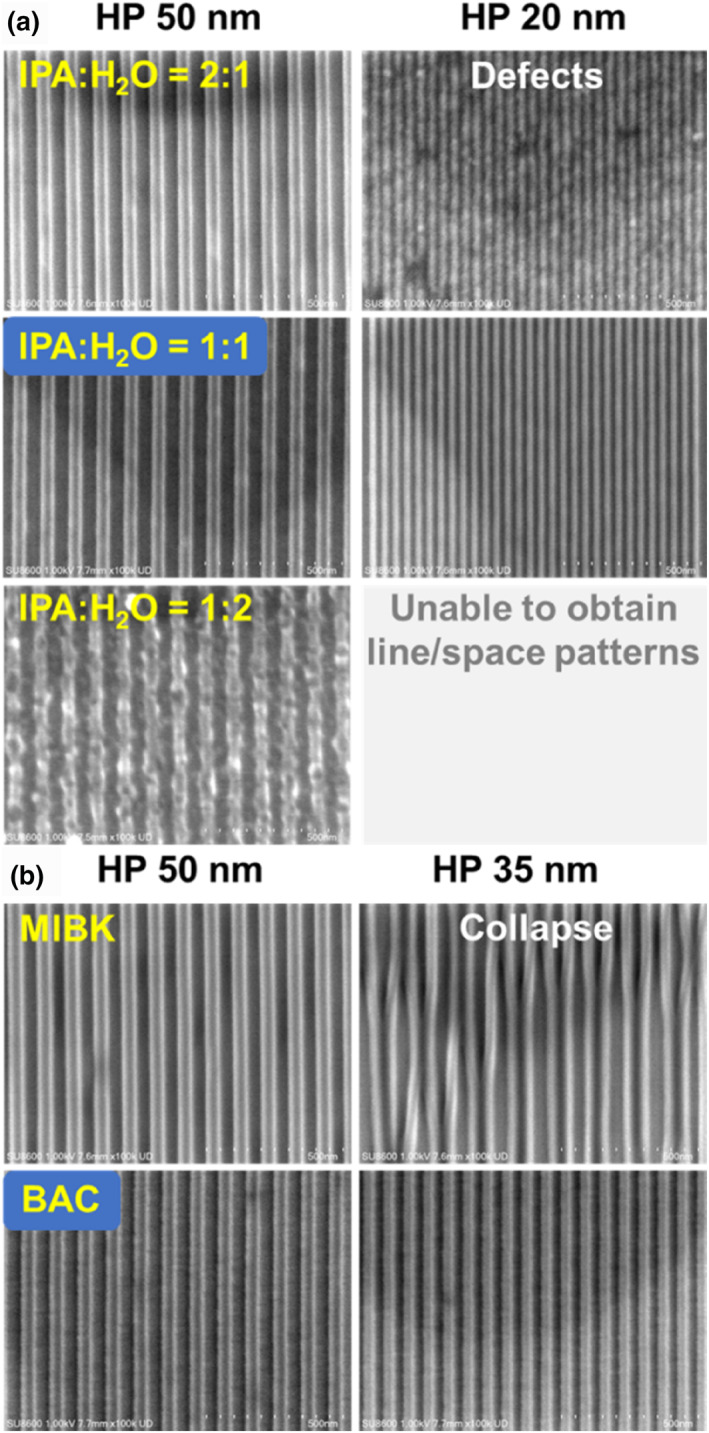
(a) Lithography patterns of X4‐I‐otfdm developed with varying IPA/H_2_O ratios. (b) Lithography patterns of X4‐NI‐tf developed with MIBK and butyl acetate.

Figure 4a demonstrates the 1:1 line/space lithographic patterns of X4‐I‐otfdm obtained in mixed developer solutions containing varying ratios of IPA and water. For HP 50 nm resolution on 45 nm film, development with IPA/H_2_O (1:2) resulted in non‐uniform linewidths, poor pattern fidelity, and severely elevated LER, with increasing IPA content, pattern fidelity exhibited progressive improvement accompanied by significantly reduced LER, achieving well‐defined lithographic patterns under both 1:1 and 2:1 (IPA:H_2_O) development conditions. Upon advancing the resolution to HP 20 nm, the experiment revealed that development with IPA:H_2_O (2:1) exhibited substantial pattern defects, elevated roughness, and degraded line‐edge morphology, whereas the IPA:H_2_O (1:1) formulation maintained superior pattern fidelity with small LER. The IPA:H_2_O 1:1 formulation was ultimately selected as the optimal developer for X4‐I‐otfdm.

X4‐NI‐tf and X4‐NI‐tfb are neutral molecules, which have high solubility in EL, PGMEA, MIBK and BAC, both exhibit poor solubility in polar solvents such as PGME, IPA and water (Table S1). Literature indicates that MIBK and BAC may serve as preferred developers for sulfoxime oxime ester‐based photoresists.^[14]^ Figure 4b compares the development performance of MIBK and BAC, demonstrating that both developers achieved well‐resolved patterns at HP 50 nm resolution. However, when the resolution was advanced to 35 nm, MIBK exhibited severe pattern collapse while BAC maintained favorable pattern fidelity with preserved structural integrity. This phenomenon arises from the pronounced capillary forces exerted by MIBK on the resist sidewalls during the development process.^[22]^ Therefore, BAC was selected as the developing agent for X4‐NI‐tf and X4‐NI‐tfb.

**FIGURE 5:**
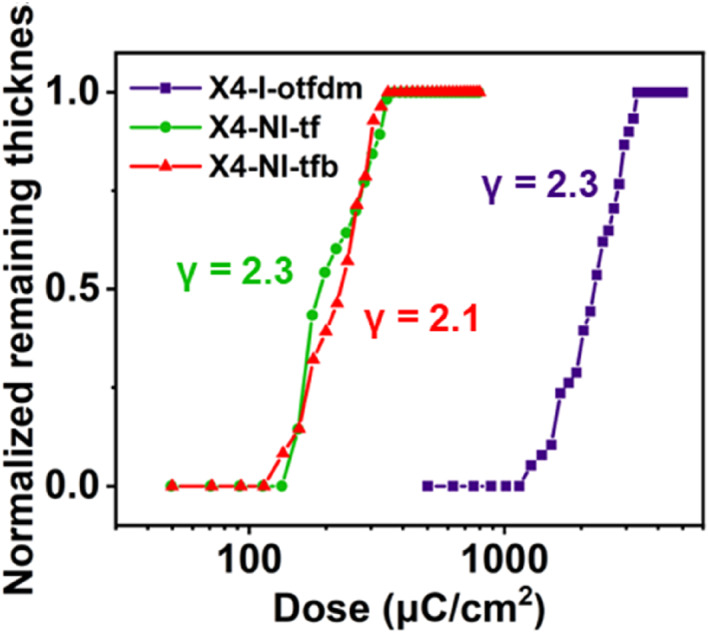
Contrast curves of X4‐I‐otfdm, X4‐NI‐tf and X4‐NI‐tfb.

The dependence of film thickness on dose provides critical information on exposure conditions, and selecting an appropriate exposure dose is critical for achieving favorable line profiles.^[23]^ As shown in Figure 5 X4‐NI‐tf and X4‐NI‐tfb exhibit significantly higher photosensitivity compared to X4‐I‐otfdm, requiring lower exposure doses to achieve equivalent retained film thickness, the doses required to achieve 100% retained film thickness (D_100_) of X4‐NI‐tf and X4‐NI‐tfb are 370 and 350 μC/cm^2^, and corresponding contrast values (*γ*) are 2.1 and 2.3, respectively, comparative analysis revealed that the introduction of bromine atoms leads slightly to a further enhancement in the electron beam exposure sensitivity of sulfoxime oxime esters. D_100_ and *γ* values of X4‐I‐otfdm are 3300 μC/cm^2^ and 2.3. The *γ* of all three components comply with industrial standards (*γ* ≥ 2.0). Experimental results demonstrate that the sensitivity of sulfoxime oxime ester surpasses that of sulfonium salts.

### 2.4 Lithographic performance of resists

**FIGURE 6:**
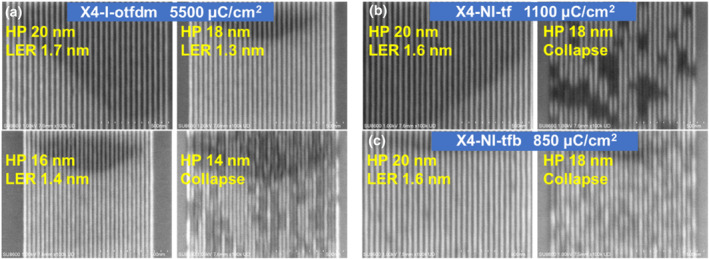
(a) The SEM images of half pitch 20, 18, 16 and 14 nm L/S patterns for X4‐I‐otfdm, film thickness: 30 nm. The SEM images of half pitch 20 and 18 nm L/S patterns for (b) X4‐NI‐tf and (c) X4‐NI‐tfb, film thickness: 25 nm.

Finally, we investigated HP 50 and 30 nm dense L/S patterns on 45 nm film (Figure S1), the ITRS stipulates that LER must remain below 8% of the critical dimension (CD). The measured LER values for X4‐I‐otfdm, X4‐NI‐tf and X4‐NI‐tfb (2.2, 2.1 and 1.9 nm) were all confirmed to conform to this standard. Reduction in film thickness facilitates the achievement of higher resolution,^[22]^ for X4‐I‐otfdm, dense L/S patterns at HP 20, 18, and 16 nm on 30 nm film were successfully achieved at ultra‐high dose of 5500 μC/cm^2^ (Figure 6a), while pattern collapse was observed at HP 14 nm, this implies that achieving higher resolution necessitates a further reduction in film thickness. For X4‐NI‐tf and X4‐NI‐tfb, the exposure doses (1100 μC/cm^2^ and 850 μC/cm^2^) required to achieve HP 20 nm resolution were substantially lower than that of X4‐I‐otfdm (5500 μC/cm^2^); the LER was 1.6 and 1.6 nm (Figure 6b,c), respectively. Notably, both X4‐NI‐tf and X4‐NI‐tfb exhibited pattern collapse at HP 18 nm.

X4‐I‐otfdm exhibits superior lithographic resolution compared to X4‐NI‐tf and X4‐NI‐tfb, a phenomenon potentially attributable to the constrained secondary electron spread range in X4‐I‐otfdm under high‐energy electron bombardment. This confinement arises from the coupled ionic interactions within its structure that restrict electron migration. In contrast, secondary electrons produced by X4‐NI‐tf and X4‐NI‐tfb lack such spatial constraints, exhibiting extended diffusion distances that result in broader post‐exposure linewidths and consequently reduced resolution.

### 2.5 Z‐factor evaluation of resists

**FIGURE 7:**
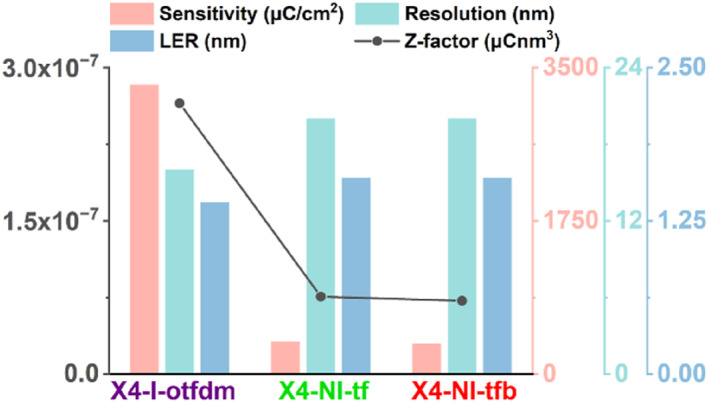
Z‐factor, sensitivity, resolution and line edge roughness of X4‐I‐otfdm, X4‐NI‐tf and X4‐NI‐tfb.

The trade‐off relationships among resolution, LER, and sensitivity govern the optimization of resist parameters and their resultant performance characteristics.^[24]^ The Z‐factor serves as a critical metric for evaluating photoresist performance, with lower Z‐values indicating superior material characteristics; the formula is expressed as follows^[25]^: *Z* = Resolution^3^ x LER^2^ x Sensitivity. Under EBL conditions, the Z‐values of X4‐I‐otfdm, X4‐NI‐tf and X4‐NI‐tfb were determined computationally as 2.65 × 10^−7^, 7.58 × 10^−8^ and 7.17 × 10^−8^ (Figure 7), respectively. Computational results demonstrate that X4‐NI‐tfb exhibits the most optimal comprehensive performance profile.

## 3 CONCLUSION

Building upon the superior rigid twisted spirobixanthene, EUV sensitive groups sulfonium salts and sulfoxime oxime esters were introduced to designed ionic nonCAR X4‐I‐otfdm and non‐ionic nonCARs X4‐NI‐tf and X4‐NI‐tfb, this design eliminates the PEB process requirement, thereby streamlining the lithographic procedure. All variants exhibit exceptional thermal stability (T_d,5%_ > 200°C) and demonstrate ideal amorphous molecular configurations by XRD, ensuring superior film‐forming capability (RMS <0.4 nm) and favorable pattern fidelity; the outcome is attributed to the superior structural characteristics of the spirobixanthene backbone. Via EBL, the sensitivity of both X4‐NI‐tf and X4‐NI‐tfb (D_100_: 370 and 350 μC/cm^2^) is significantly higher than that of X4‐I‐otfdm (D_100_: 3300 μC/cm^2^). X4‐NI‐tf and X4‐NI‐tfb achieves HP 20 nm, LER 1.6 nm dense L/S patterns on 25 nm film, X4‐I‐otfdm achieves HP 16 nm, LER 1.4 nm dense L/S patterns on 30 nm film, however, the excessively low electron beam sensitivity resulted in the Z‐factor lower than those of X4‐NI‐tf and X4‐NI‐tfb. This work provides the outstanding examples for nonchemically amplified MGRs.

## CONFLICT OF INTEREST STATEMENT

The authors declare no conflicts of interest.

## ETHICS STATEMENT

No animal or human experiments were involved in this study.

## Supporting information

Supporting Information S1

## Data Availability

The data that support the findings of this study are available on request from the corresponding author. The data are not publicly available due to privacy or ethical restrictions.
