# Supplementary material for: Molecular nonchemically amplified resists based on spirobixanthene backbone: Sulfoxime oxime esters versus sulfonium salts
Source: Smart Mol. 2025 Nov 24;4(1):e70028. doi: 10.1002/smo2.70028 (PMC13104132; doi:10.1002/smo2.70028)
Supplement: Supplementary file 1 — Supporting Information S1 [file SMO2-4-e70028-s001.docx]

Supporting Information

Molecular Nonchemically Amplified Resists Based on Spirobixanthene Backbone: Sulfoxime Oxime Esters *vs* Sulfonium Salts

Yu Yan, Chenfei Zhao, Jingwen Hui, Xinfu Zhang,* Xue Zhang, Pengzhong Chen, Xiaojun Peng,* and Yi Xiao*

State Key Laboratory of Fine Chemicals, Frontiers Science Center for Smart Materials Oriented Chemical Engineering, Dalian University of Technology, Dalian 116024, China.

E-mail: zhangxinfu@dlut.edu.cn; pengxj@dlut.edu.cn; xiaoyi@dlut.edu.cn

**Experimental Section**

**Chemicals and Materials:** All the standard reagents and chemicals were purchased from commercial sources and used without any further purification.

**Instrumentations:** Nuclear magnetic resonance (NMR) analysis was performed on Bruker AVANCE NEO 600M. Thermogravimetry Analysis (TGA) measurements were performed on TA Q500 with heating rate of 10 °C/min from rt to 600 °C in N_2_ atmosphere. Differential scanning calorimetry (DSC) measurements were performed on TA Q20 with heating rate of 10 °C/min from rt to 120 °C in N_2_ atmosphere. X-ray diffraction (XRD) was tested on Rigaku SmartLab 9kW. Matrix-assisted laser desorption/ionization mass spectrometry (MALDI-MS) data were tested on Bruker Ultraflexetreme. The film thickness and surface roughness were measured by atomic force microscopy (AFM) Bruker JPK Nanowizard 4XP. E-beam lithography (EBL) was performed on HITACHI SU8600 with Raith Controller. Line width (LW) and line edge roughness (LER) are calculated by open software SMILE.^[1]^

**Patterning Processes of Resist**

X4-I-otfdm (20 mg) was dissolved in 1 mL ethyl lactate (EL) to get a resist solution, X4-NI-tf or X4-NI-tfb (20 mg) was dissolved in 1 mL 1-methoxy-2-propyl acetate (PGMEA) to get a resist solution. The solution was filtered by 0.22 μm Nylon66 springe filter, then stored in a brown bottle away from light. The 25 μL solution was spin coated on a 10 mm x 10 mm square wafer at 2000 – 4500 rpm for 30 s to give film thickness ranged from 45 nm to 25 nm, the wafer was exposed by EBL tool (SU8600 + Raith). The expose dose was set with acceleration voltage 30 keV and beam current 30 pA. After exposure, the wafer of X4-I-otfdm was developed directly in mixed solution of IPA/H_2_O (v/v = 1/1) for 30 s at room temperature, the wafer of X4-NI-tf or X4-NI-tfb was developed directly in BAC for 30 s at room temperature. Finally, the wafer was dried with a stream of nitrogen.

**Contrast Curve of Resist**

The 25 μL solution was spin coated on a 10 mm x 10 mm square wafer at 1500 rpm for 30 s to give film thickness. The acceleration voltage and beam current were set to 30 keV and 30 pA, respectively, then a 6 × 6 array of 2 μm × 2 μm test patterns was e-beam exposed with doses form low value to high value (Figure S1. Incremental mode: add). After exposure, the wafer of X4-I-otfdm was developed directly in mixed solution of IPA/H_2_O (v/v = 1/1) for 30 s at room temperature, the wafer of X4-NI-tf or X4-NI-tfb was developed directly in BAC for 30 s at room temperature. Finally, the wafer was dried with a stream of nitrogen. The film thickness of each 2 μm × 2 μm square region was measured using AFM, and the data were normalized. A contrast curve was generated by plotting the normalized film thickness against the dose, with dose as the abscissa and normalized thickness as the ordinate. The contrast (γ) of resist was calculated using the following formula:

$$\gamma= {(log\frac{D_{100}}{D_{0}})}^{-1}$$

D_100_ represents the minimum dose at which resist thickness ceases to change, D_0_ denotes the maximum dose at which resist is completely removed.

**Table S1.** The solubility of X4-I-otfdm, X4-NI-tf and X4-NI-tfb in different solvent. Concentration: 20 mg/mL

| Solvent | X4-I-otfdm | X4-NI-tf | X4-NI-tfb |
| --- | --- | --- | --- |
| EL | ++ | ++ | ++ |
| PGMEA | -- | ++ | ++ |
| PGME | ++ | -- | -- |
| MIBK | -- | ++ | ++ |
| IPA | ++ | -- | -- |
| BAC | -- | ++ | ++ |
| H_2_O | ++ | -- | -- |

++: soluble --: insoluble


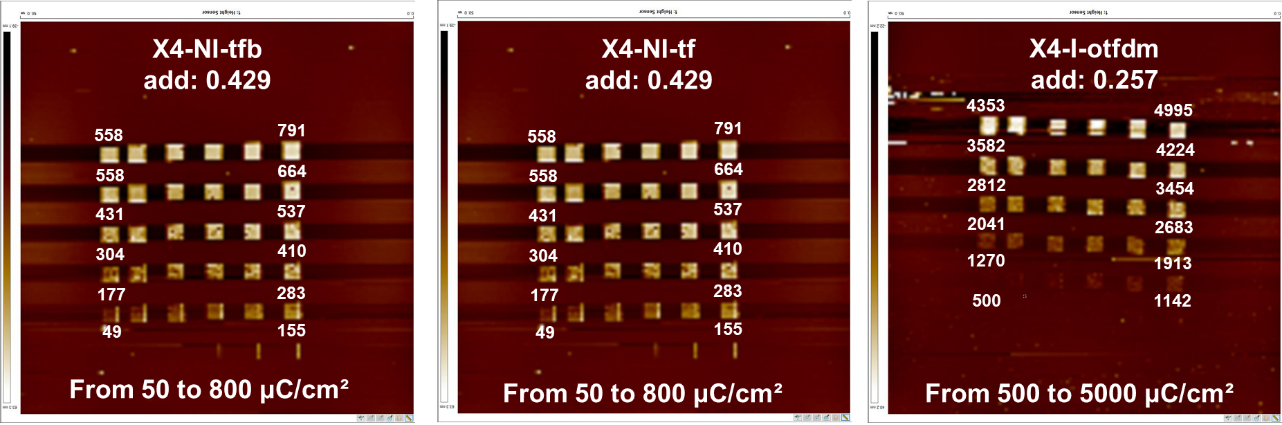


**Figure S1.** AFM images of the variation of film thickness for X4-NI-tfb, X4-NI-tf and X4-I-otfdm.

**
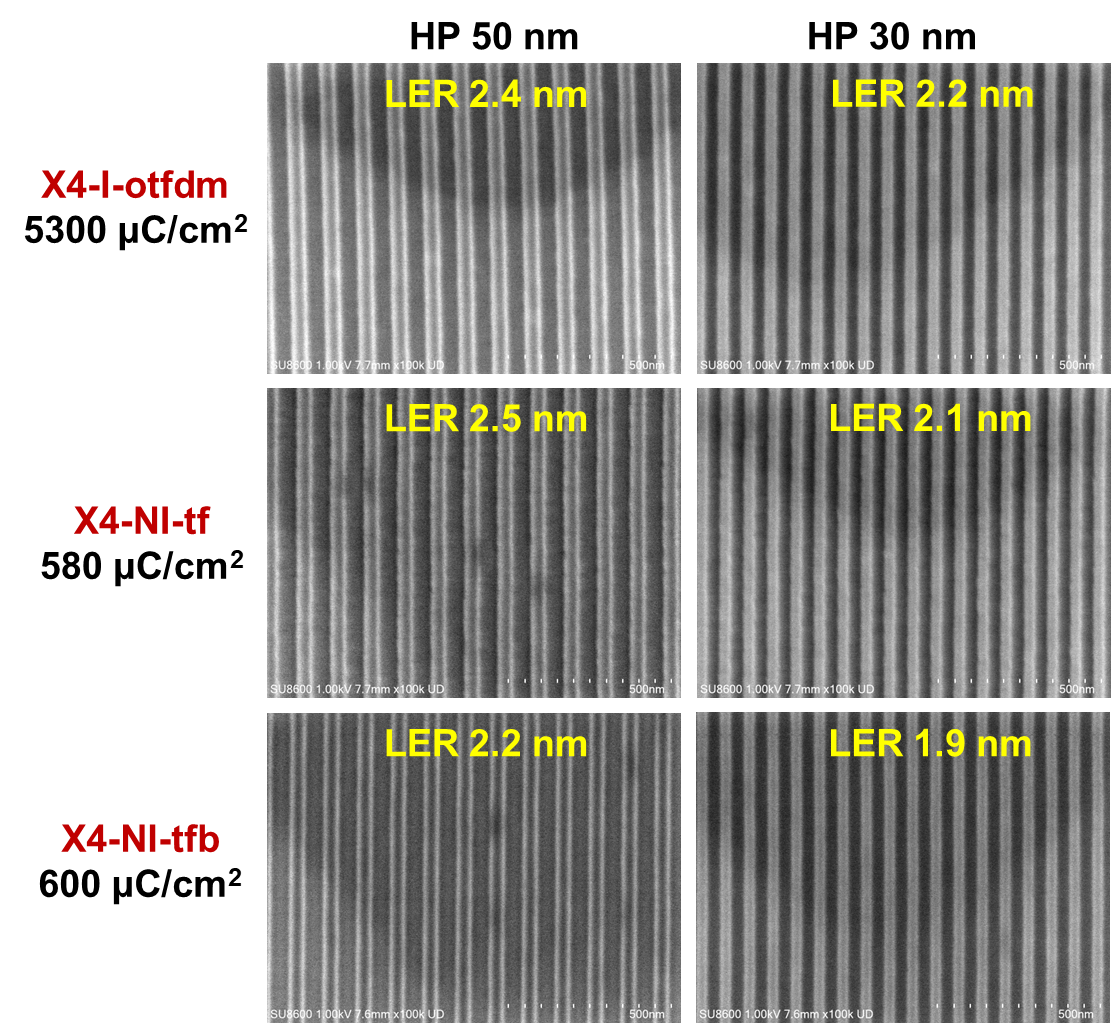
**

**Figure S2.** The SEM images of HP 50 and 30 nm L/S patterns of X4-I-otfdm, X4-NI-tf and X4-NI-tfb.

Synthesis of X4-I-otfdm, X4-NI-tf and X4-NI-tfb

**2,2',7,7'-tetrakis(4-(methylthio)phenyl)-9,9'-spirobi[xanthene] (a):** Under Ar, the mixture of 2,2',7,7'-tetrabromo-9,9'-spirobi[xanthene] (500 mg, 753.03 μmol), (4-(methylthio)phenyl)boronic acid (760 mg, 4.52 mmol), K_2_CO_3_ (1.25 g, 9.04 mmol) in THF/H_2_O (18 mL, v/v = 5:1) was warmed to 80 ℃, then Pd(OAc)_2_ (34 mg, 150.61 μmol), SPhos (124 mg, 301.21 μmol) was added, the mixture was stirred at 80 ℃ for 9 h. Cooled to rt, diluted with DCM, washed with NaCl aq, filtered through a pad of celiteto, the solvent was removed away under reduced pressure, the residue was purified by recrystallization with DCM/PE (v/v = 1:4) to give 2,2',7,7'-tetrakis(4-(methylthio)phenyl)-9,9'-spirobi[xanthene] (611 mg, 97%) as a white solid. ^1^H NMR (400 MHz, Chloroform-*d*) δ 7.41 (dd, *J* = 8.5, 2.2 Hz, 4H), 7.28 (s, 2H), 7.25 (s, 2H), 7.25 – 7.21 (m, 8H), 7.21 – 7.17 (m, 8H), 7.13 (d, *J* = 2.2 Hz, 4H), 2.44 (s, 12H). ^13^C NMR (151 MHz, Chloroform-*d*) δ 148.91, 137.45, 137.13, 136.28, 130.39, 129.67, 129.59, 127.19, 127.01, 126.92, 116.96, 43.36, 16.04. HRMS m/z: calcd for C_53_H_40_O_2_S_4_ [M] 836.1911; found: 836.1876

**(9,9'-spirobi[xanthene]-2,2',7,7'-tetrayltetrakis(benzenene-4,1-diyl))tetrakis(dimethylsulfonium) trifluoromethanesulfonate (X4-I-otfdm):** Under Ar, iodomethane (270 μL, 4.30 mmol) was added dropwise to a solution of 2,2',7,7'-tetrakis(4-(methylthio)phenyl)-9,9'-spirobi[xanthene] (300 mg, 358.36 μmol) and CF_3_SO_3_Ag (405 mg, 1.58 mmol) in CH_3_CN (15 mL) in the dark at 0 ℃, the mixture was stirred at rt for 24 h. Filtered and washed with acetonitrile, the collected filtrates were concentrated in a rotary evaporator and the resultant crude product was purified by washing several times with Et_2_O to give (9,9'-spirobi[xanthene]-2,2',7,7'-tetrayltetrakis(benzenene-4,1-diyl))tetrakis(dimethylsulfonium) trifluoromethanesulfonate (420 mg, 78%) as a white solid. ^1^H NMR (600 MHz, DMSO-*d*_6_) δ 8.02 – 7.99 (m, 8H), 7.79 (dd, *J* = 8.7, 2.3 Hz, 4H), 7.68 – 7.65 (m, 8H), 7.54 (d, *J* = 8.6 Hz, 4H), 7.25 (d, *J* = 2.3 Hz, 4H), 3.49 (s, 24H). ^13^C NMR (151 MHz, DMSO-*d*_6_) δ 149.26, 143.64, 134.05, 131.84, 130.57, 129.23, 127.94, 127.85, 125.25, 121.75, 119.61, 117.71, 42.76, 28.27.

**4,4',4'',4'''-(9,9'-spirobi[xanthene]-2,2',7,7'-tetrayl)tetrabenzenesulfonyl fluoride (b):** Under Ar, the mixture of 2,2',7,7'-tetrabromo-9,9'-spirobi[xanthene] (500 mg, 753.03 μmol), (4-(fluorosulfonyl)phenyl)boronic acid (922 mg, 4.52 mmol), anhydrous K_3_PO_4_ (1.6 g, 7.53 mmol) in THF/H2O (18 mL, v/v = 5:1) was warmed to 85 ℃, then Pd(OAc)_2_ (34 mg, 150.61 μmol), SPhos (124 mg, 301.21 μmol) was added, the mixture was stirred at 85 ℃ for 10 h. Cooled to rt, diluted with DCM, washed with NaCl aq, filtered through a pad of celiteto, the solvent was removed away under reduced pressure, the residue was purified by recrystallization with DCM/t-BuOH (v/v = 1:4) to give 2,2',4,4',5,5',7,7'-octakis(4-(methylthio)phenyl)-9,9'-spirobi[xanthene] (680 mg, 92%) as a white solid. ^1^H NMR (500 MHz, Chloroform-d) δ 7.97 – 7.93 (m, 8H), 7.56 (dd, J = 8.6, 2.3 Hz, 4H), 7.53 (d, J = 8.5 Hz, 8H), 7.44 (d, J = 8.6 Hz, 4H), 7.18 (d, J = 2.3 Hz, 4H). ^13^C NMR (126 MHz, Chloroform-d) δ 149.86, 147.03, 135.00, 131.81, 131.61, 130.28, 129.55, 129.22, 129.16, 128.18, 127.82, 118.07, 43.34. MS m/z: calcd for C_49_H_28_F_4_O_10_S_4_ [M - H] 979.08; found: 979.1

**tetrayltetrakis(benzenenesulfonyl))tetrakis(2,2,2-trifluoro-1-phenyl-2,2,2-trifluoro-1-phenylethan-1-one oxime) (X4-NI-tf):** Under argon atmosphere, the mixture of 4,4',4'',4'''-(9,9'-spirobi[xanthene]-2,2',7,7'-tetrayl)tetrabenzenesulfonyl fluoride (500 mg, 509.69 μmol), HMDS (531 μL, 2.55 mmol), BTMG (4 μL, 20.39 μmol) and (E)-2,2,2-trifluoro-1-phenylethan-1-one oxime (482 mg, 2.55 mmol) in anhydrous acetonitrile/DCM (20 mL/10 mL) was stirred at rt for 3 h. The solvent was concentrated in vacuo and the residue was purified by chromatography (silica gel, PE: EA = 5:1) to give (1E,1'E,1''E,1'''E)-O,O',O'',O'''-(9,9'-spirobi[xanthene]-2,2',7,7'-tetrayltetrakis(benzenenesulfonyl))tetrakis(2,2,2-trifluoro-1-phenyl-2,2,2-trifluoro-1-phenylethan-1-one oxime) (700 mg, 83%) as a white solid. ^1^H NMR (500 MHz, Chloroform-d) δ 7.94 (d, J = 8.2 Hz, 8H), 7.58 (dd, J = 8.8, 2.1 Hz, 4H), 7.51 (d, J = 8.0 Hz, 12H), 7.45 (q, J = 8.5, 8.0 Hz, 12H), 7.37 (d, J = 7.7 Hz, 8H), 7.22 (d, J = 2.1 Hz, 4H). ^13^C NMR (126 MHz, Chloroform-d) δ 154.36, 149.71, 146.21, 135.18, 132.87, 131.86, 130.28, 129.97, 129.59, 128.98, 128.50, 128.14, 128.00, 127.65, 127.41, 124.64, 120.80, 118.60, 117.92, 43.33. MS m/z: calcd for C_81_H_48_F_12_N_4_O_14_S_4_ [M + Na] 1679.18; found: 1679.4

**(1E,1'E,1''E,1'''E)-O,O',O'',O'''-(9,9'-spirobi[xanthene]-2,2',7,7'-tetrayltetrakis(benzenenesulfonyl))tetrakis(1-(4-bromophenyl)-2,2,2-trifluoro-1-(4-bromophenyl)-2,2,2-trifluoroethan-1-one oxime) (X4-NI-tfb):** Under Ar, the mixture of 4,4',4'',4'''-(9,9'-spirobi[xanthene]-2,2',7,7'-tetrayl)tetrabenzenesulfonyl fluoride (500 mg, 509.69 μmol), HMDS (531 μL, 2.55 mmol), BTMG (4.11 μL, 20.39 μmol) and (E)-1-(4-bromophenyl)-2,2,2-trifluoroethan-1-one oxime (683 mg, 2.55 mmol) in anhydrous acetonitrile/DCM (21 mL, v/v = 2:1) was stirred at rt for 3 h. The solvent was concentrated in vacuo and the residue was purified by chromatography (silica gel, PE: EA = 5:1) to give (1E,1'E,1''E,1'''E)-O,O',O'',O'''-(9,9'-spirobi[xanthene]-2,2',7,7'-tetrayltetrakis(benzenenesulfonyl))tetrakis(1-(4-bromophenyl)-2,2,2-trifluoro-1-(4-bromophenyl)-2,2,2-trifluoroethan-1-one oxime) (400 mg, 40%) as a white solid. ^1^H NMR (500 MHz, DMSO-*d*_6_) δ 7.94 (dt, *J* = 6.4, 4.4 Hz, 8H), 7.86 – 7.64 (m, 20H), 7.58 – 7.52 (m, 4H), 7.41 (dd, *J* = 11.9, 8.0 Hz, 8H), 7.33 – 7.24 (m, 4H). ^13^C NMR (151 MHz, DMSO-*d*_6_) δ 153.59, 149.19, 145.29, 132.20, 132.03, 131.42, 130.95, 130.36, 129.61, 128.17, 127.46, 126.30, 125.97, 122.83, 119.91, 118.07, 117.83, 42.59, 26.30.

^1^H NMR and HRMS of molecules


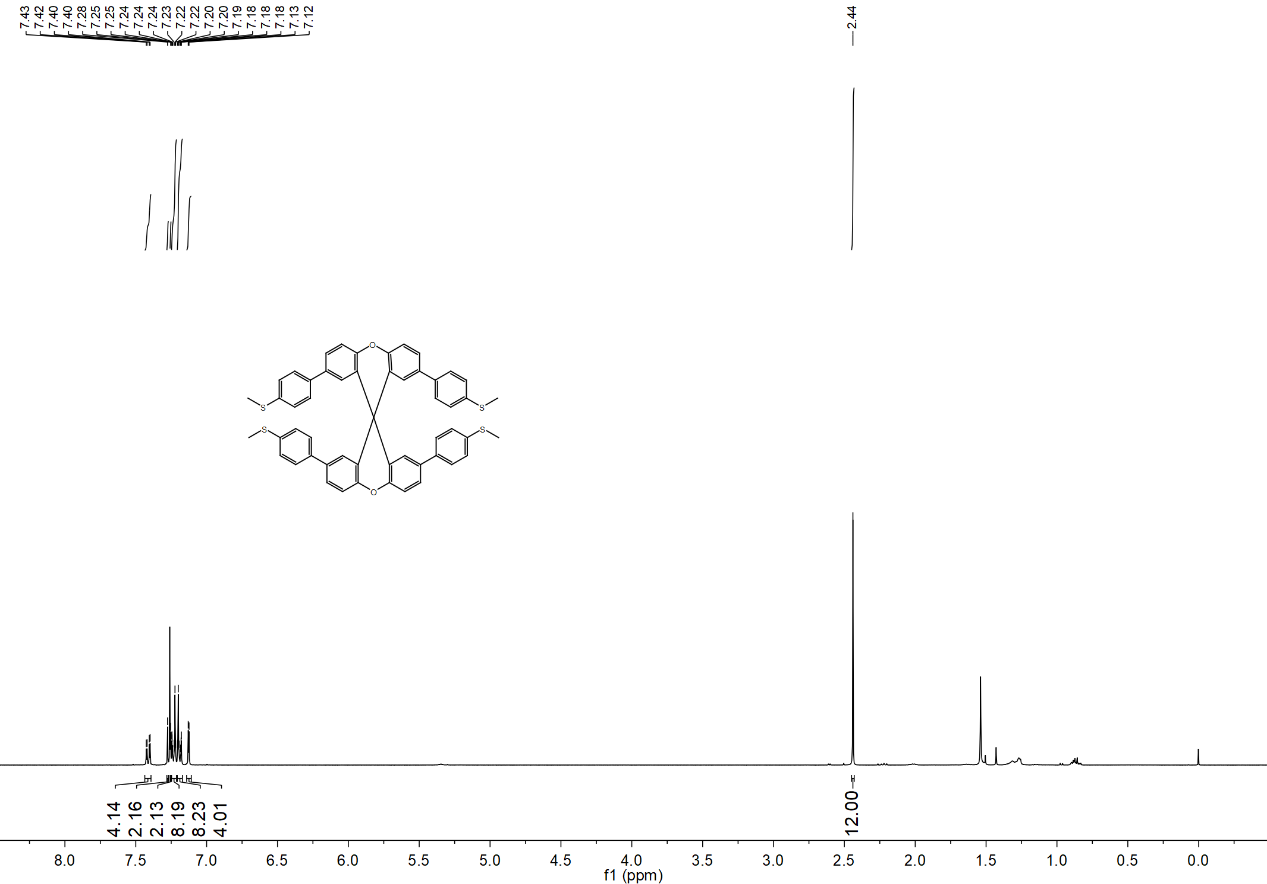


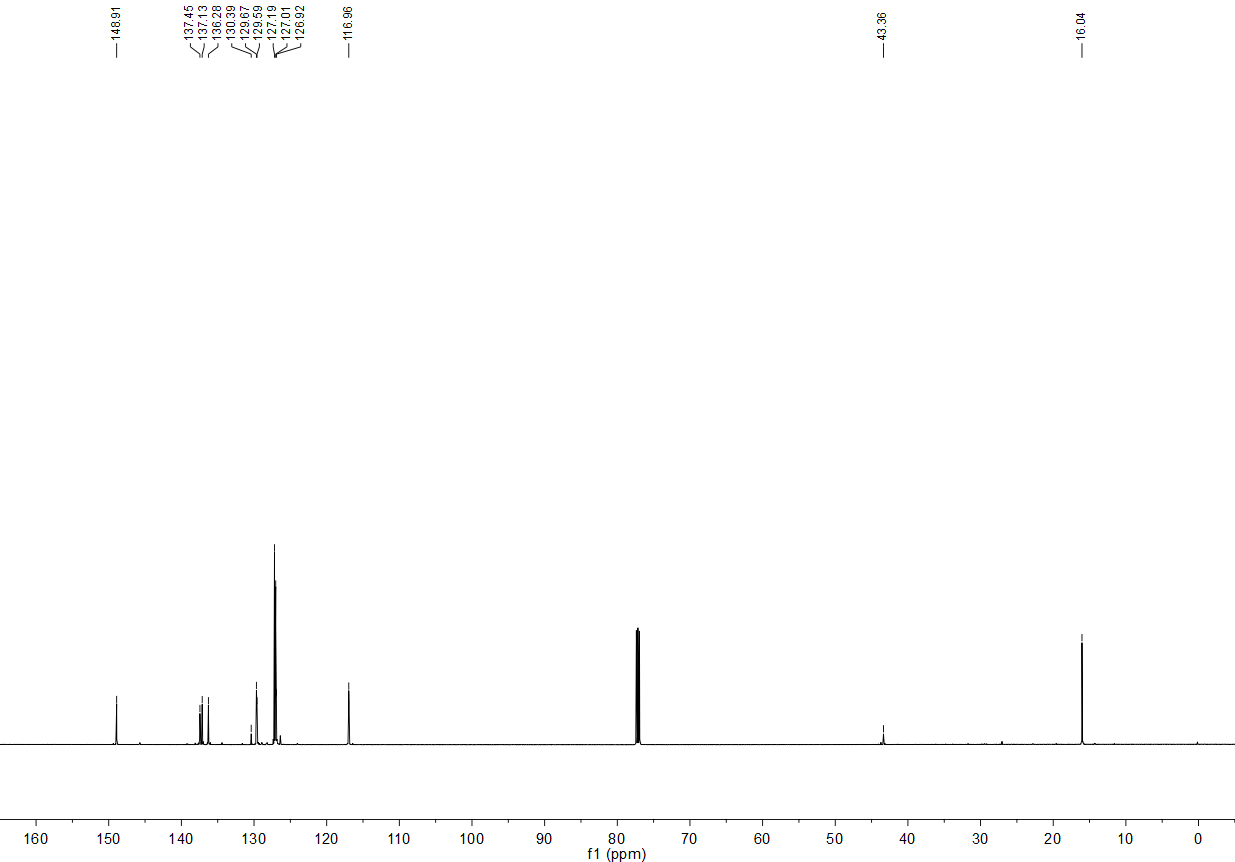

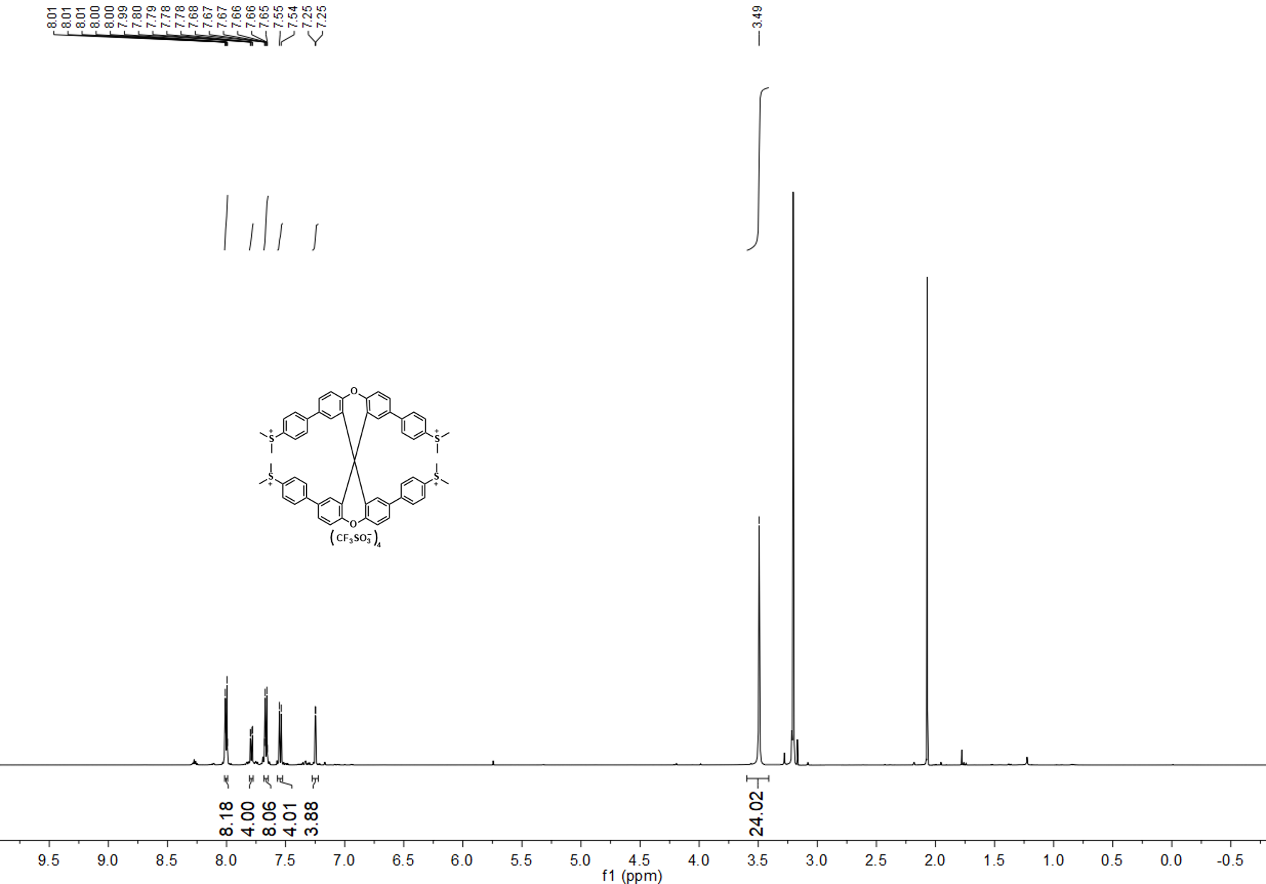


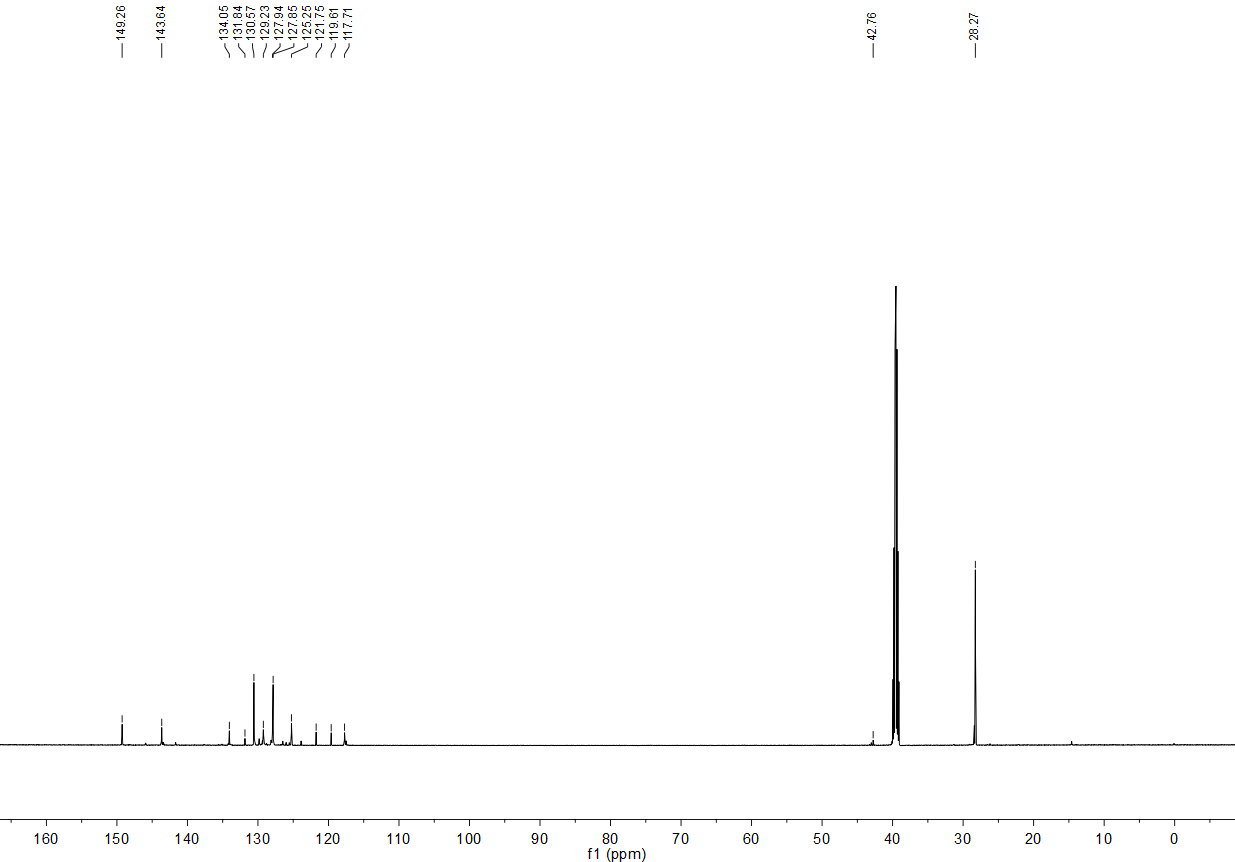


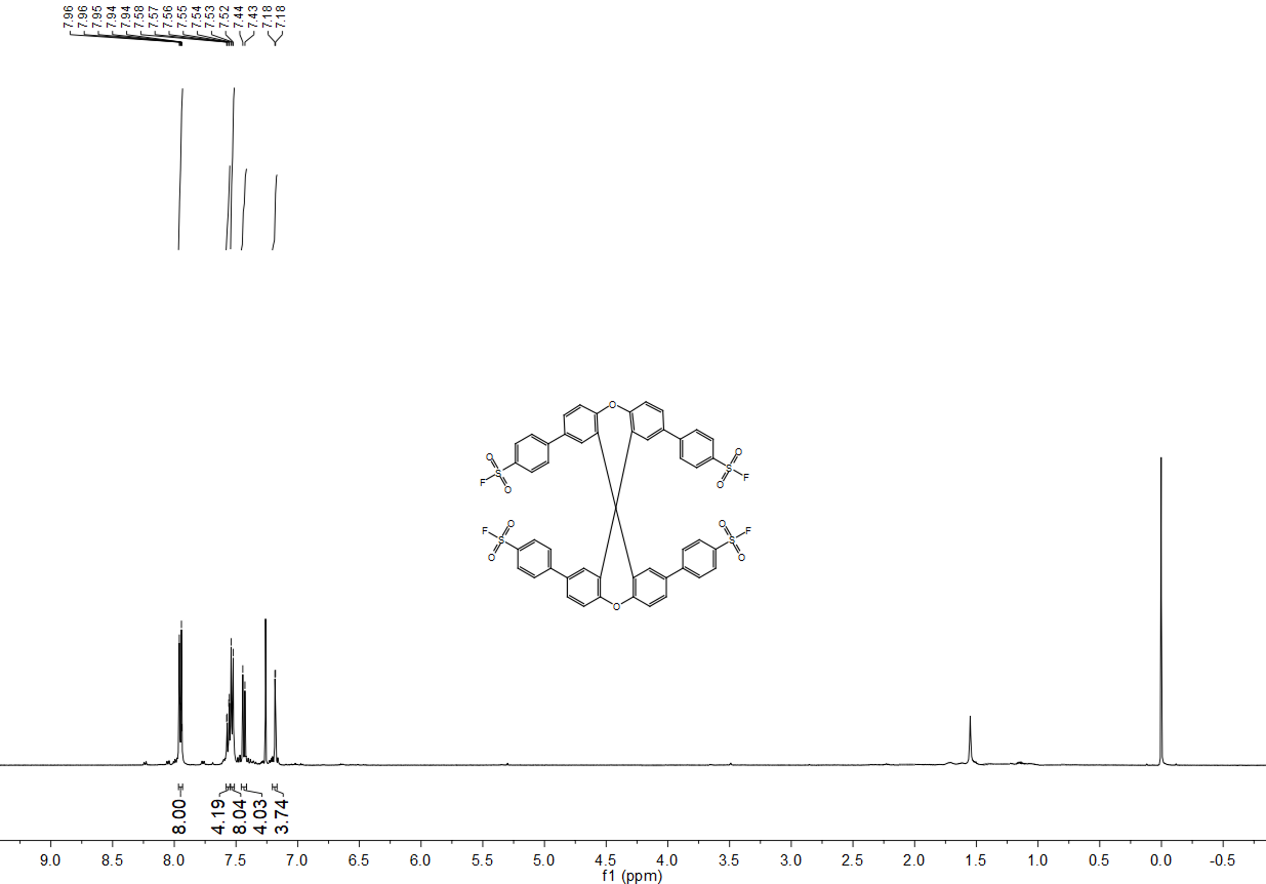


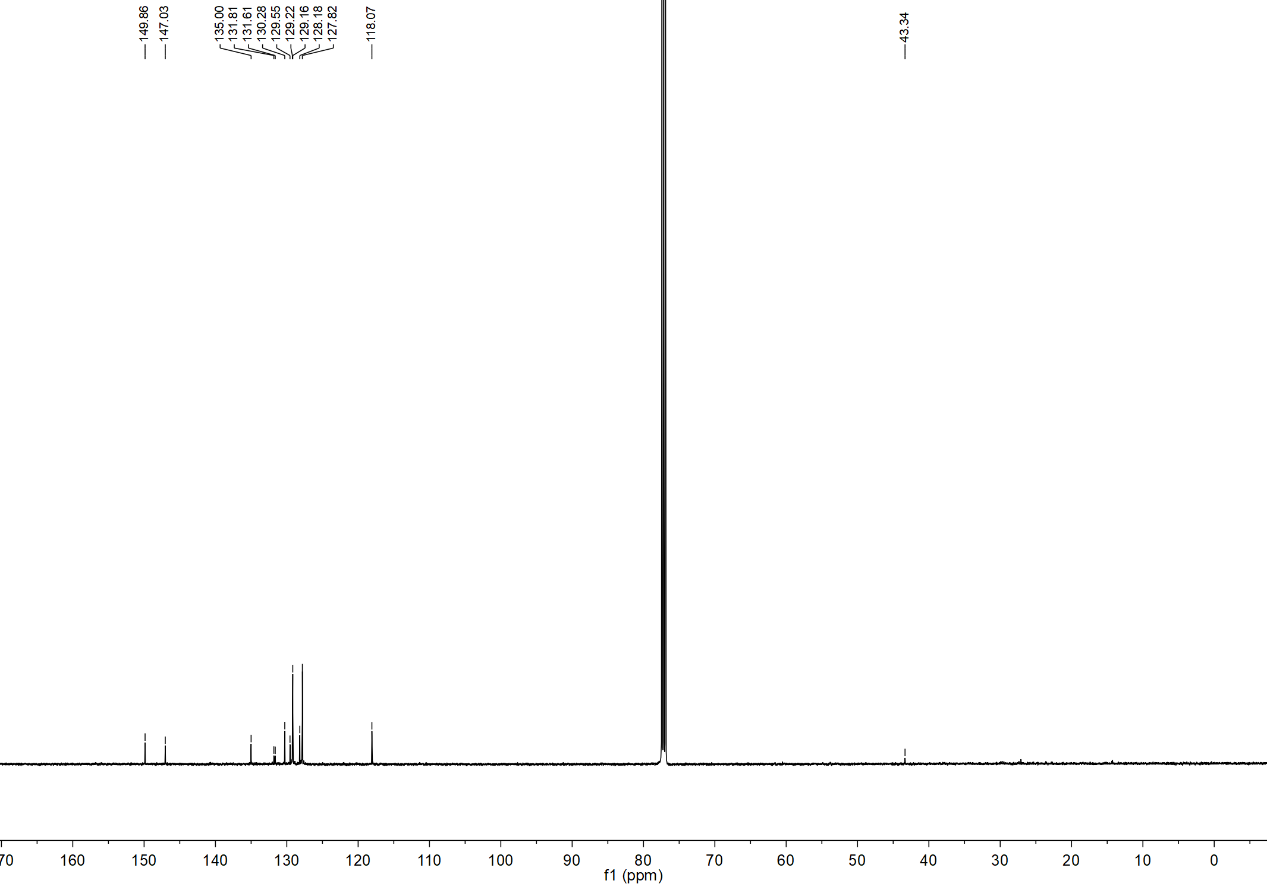

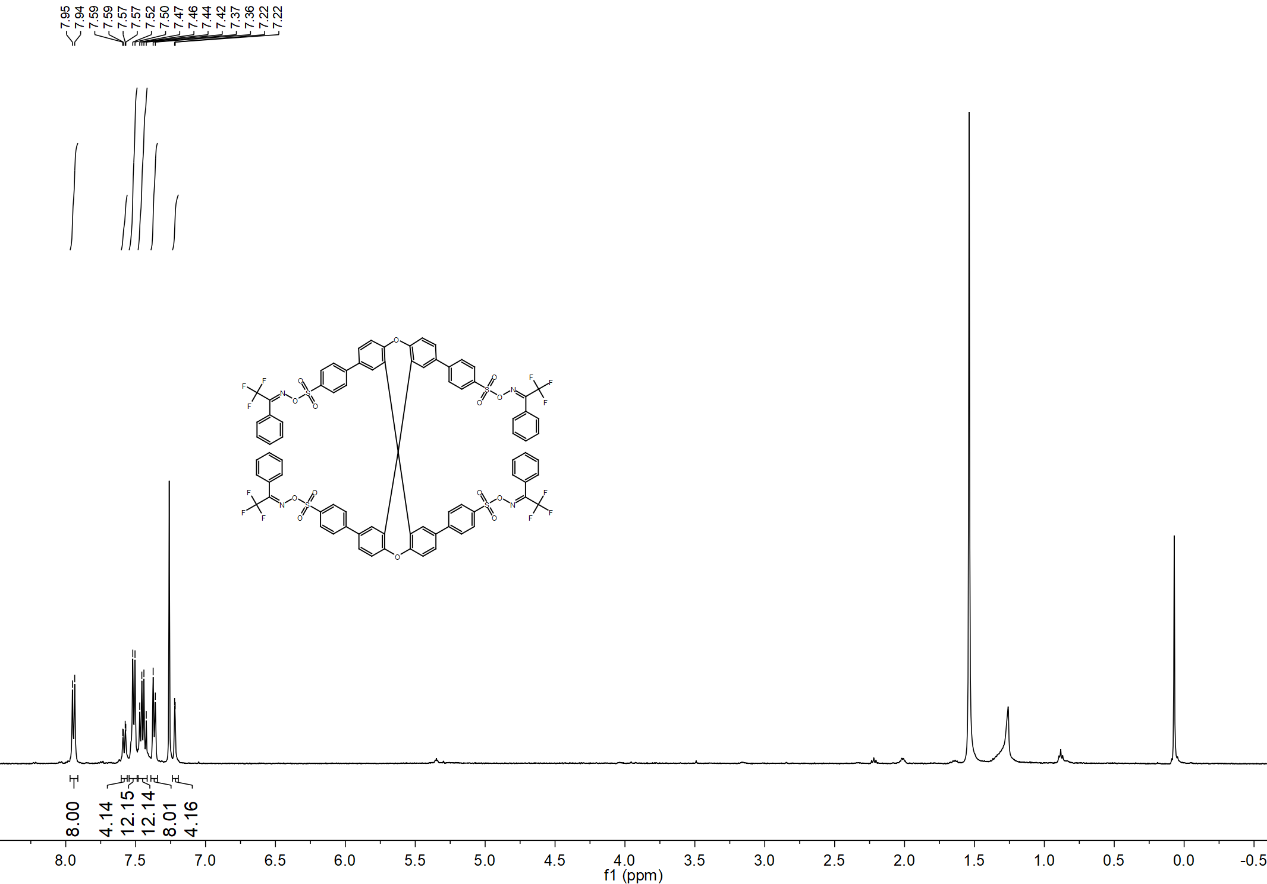


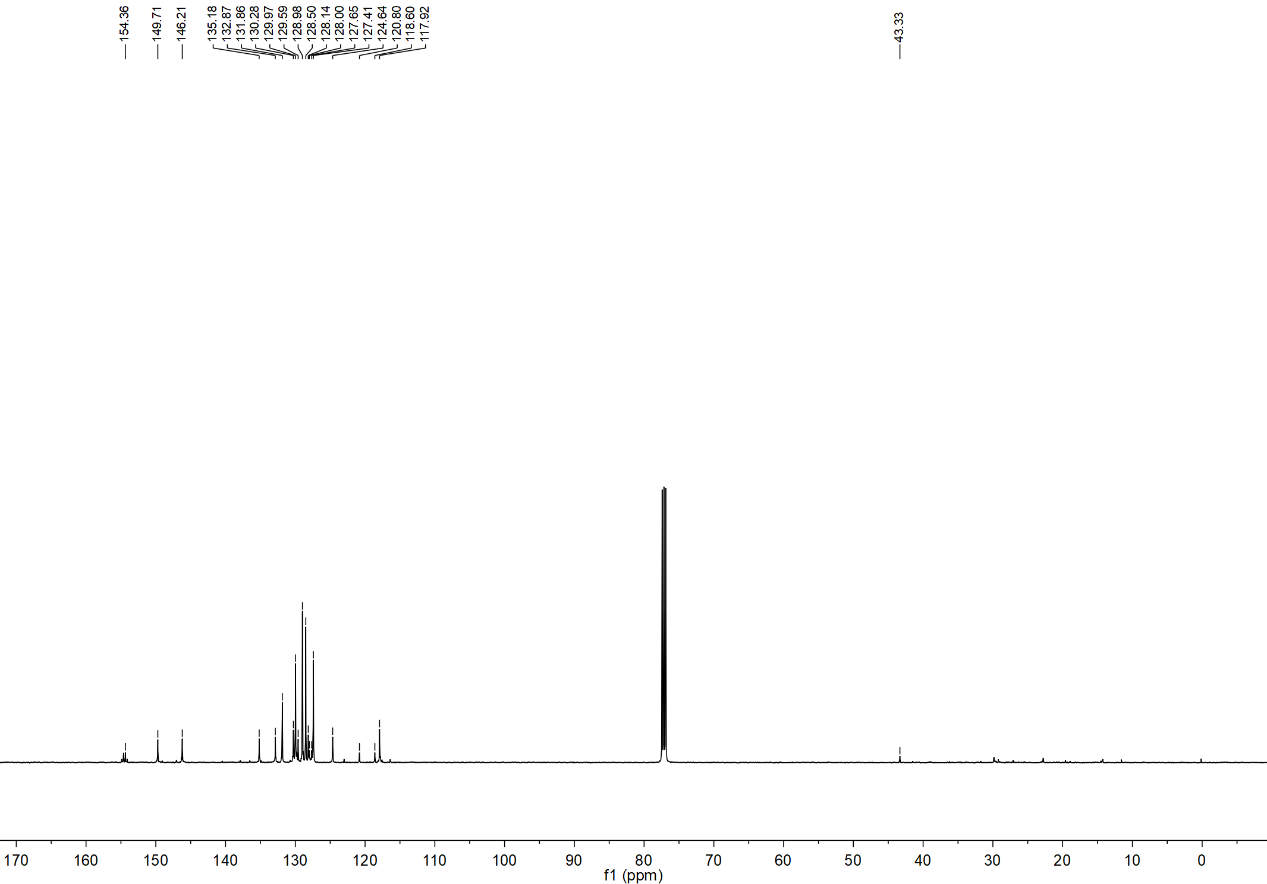

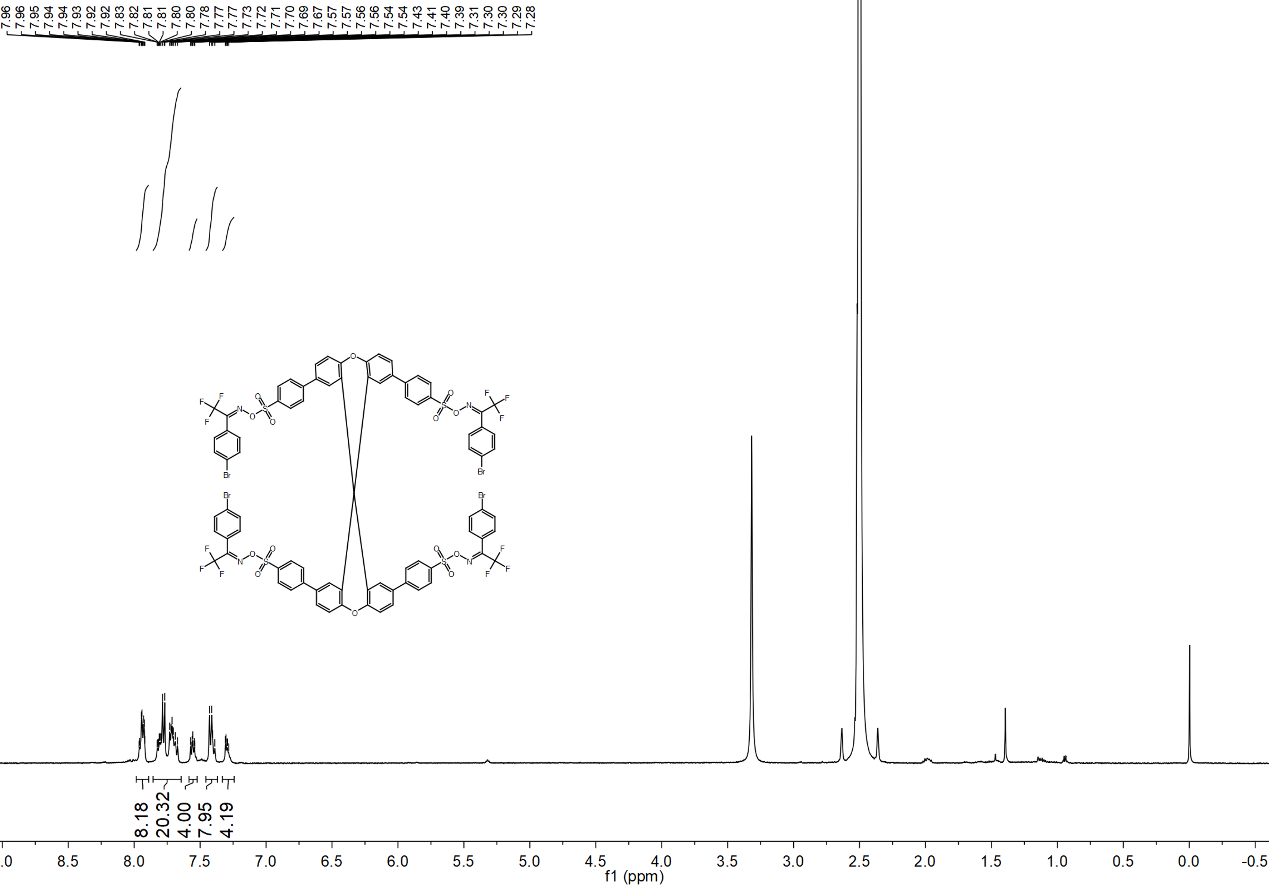


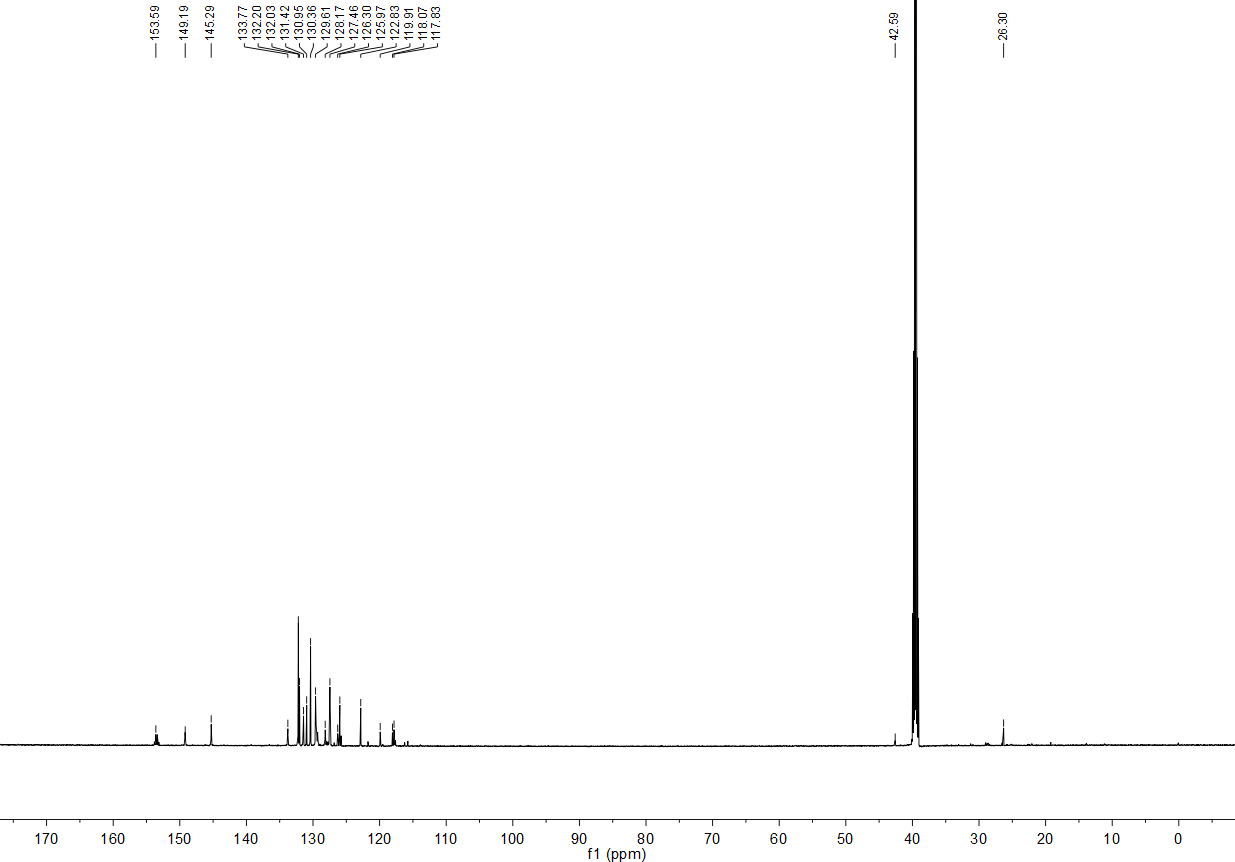


**Reference**

[1] I. Mochi, M. Vockenhuber, T. Allenet, Y. Ekinci, In Photomask Technology 2021 2021.
